# Paucity of gastrointestinal plasma cells in common variable immunodeficiency

**DOI:** 10.1097/ACI.0000000000001040

**Published:** 2024-10-07

**Authors:** Jan Willem N. Marsden, Miangela M. Laclé, Mirjam Severs, Helen Louisa Leavis

**Affiliations:** a University Medical Center Utrecht, Department of Clinical Immunology and Rheumatology; b University Medical Center Utrecht, Department of Pathology, Utrecht University, Utrecht; c Radboud University Medical Center Nijmegen, Department of Gastroenterology, Nijmegen, The Netherlands

**Keywords:** common variable immunodeficiency, enteropathy, histopathology, immunoglobulin-A, plasma cells

## Abstract

**Purpose of review:**

Common variable immunodeficiency enteropathy (CVID-E) is a noninfectious complication of CVID caused by chronic inflammation of the gastrointestinal (GI) tract. Based on literature, a paucity or lack of plasma cells, although not obligatory for diagnosis, is a pathognomonic feature of CVID and more frequent in CVID-E. However, there is no consensus on standardized histopathological analysis of this feature in biopsies. In this systematic review, we highlight methods of reproducible plasma cell quantification of biopsies in CVID and describe the plasma cell counts and classes as presented in the literature.

**Recent findings:**

Reduced plasma cell counts are commonly found over the entire GI tract, except for in the oesophagus. Immunoglobulin A^+^ (IgA^+^) plasma cells appear to be the most commonly reduced plasma cell class in CVID, yet there is scarce literature on the predictive value of low IgA^+^ plasma cell counts in CVID-E.

**Summary:**

We propose two optimized methodologies of quantification using a cut-of value of <10 plasma cells per HPF at 40× magnification, or a proportion of ≥1–5% of total mononuclear cells, recorded over ≥3 sections, and in ≥2 biopsies, as the most conservative agreeable definitions for a paucity of plasma cells to be used in diagnostics and further research.

## INTRODUCTION

Common variable immunodeficiency (CVID) is the most common symptomatic primary immunodeficiency, affecting about 1 in every 25 000 Caucasians [1]. These patients have a marked reduction in serum levels of both immunoglobulin G (IgG) and either immunoglobulin A (IgA) or immunoglobulin A (IgM) as a result of a B-cell dysfunction [2]. Burden of disease is high in CVID, the annual mean rate of disability adjusted life years (DALY) in the cohort associated with the European Society for Immunodeficiencies (ESID) was 36 785 [3]. This ranks CVID in the top 10 of diseases with the highest individual burden. CVID can result in infectious and noninfectious inflammatory complications [4]. CVID patients with inflammatory complications have even lower quality of life and significantly higher mortality rates than those with only infections [5,6].

One of the most affected organs in CVID is the gastrointestinal (GI)-tract, with studies reporting ranges between 20% and 35% of CVID patients experiencing noninfectious GI disease or CVID-E [7–9]. Manifestations of CVID-E are heterogeneous and can be grouped in the following conditions: Chronic gastritis, villous atrophy, chronic bacterial overgrowth, chronic exudative enteropathy, chronic enteritis, and microscopic colitis [10]. The first line of treatment for CVID-E usually consists of enteral steroids such as budesonide. Other immunosuppressive therapies have been used with variable and inconsistent results, and there is no consensus on the optimal method of evaluation and research on treatment effect [10].

Currently, the role of histological assessment of CVID-E serves to exclude other causes of chronic gastrointestinal diseases, such as celiac disease, graft-vs-host disease, and various infections such as Norovirus or Giardia [11]. The paucity of plasma cells is frequently noted as the most significant histological feature within the histopathology of CVID-E [2,4,12–14], with studies reporting between 50% and 90% of patients who have undergone biopsies exhibiting an absence of plasma cells [15,16,17^▪^,18^▪^]. The role of this paucity of plasma cells in the GI-tract in the pathogenesis of CVID-E is unknown. However, it has been theorized that a paucity in IgA plasma cells contributes to a dysfunctional barrier function in the gut [19^▪▪^,^20^]. The definition of paucity in this case varies between studies, and paucity and absence are sometimes combined into one statistic. Furthermore, the number of biopsies, their location, and the number of sections which must be analysed for the assessment of plasma cell paucity is unclear.

In this systematic review, we aim to explore the best method to characterize and classify the plasma cell counts in the GI tract of CVID patients based on current literature, and to propose standardized recommendations of plasma cell assessment in the GI tract as a basis for further diagnostic and research purposes. Standardized assessment of plasma cell counts may contribute towards a better definition of CVID-E, because this feature is considered to be pathognomonic to the disease. Using a systematic approach, we will focus on the methods employed to quantify plasma cell count, optimal location(s), number of slides to analyse, classes of plasma cells, and number of biopsies to draw solid conclusions on plasma cell counts. 

**Box 1:**
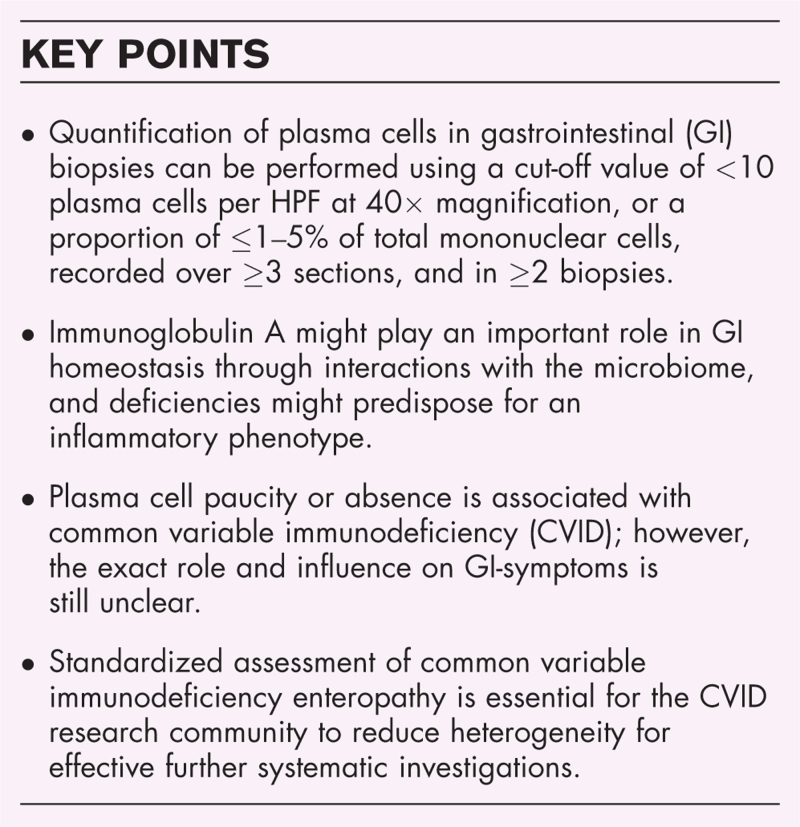
no caption available

## METHODS

To ensure reproducibility and minimal bias in the formulation of the search, the construction of our search string was done according to a specific methodology as developed by Bramer *et al.*[21]. We started by constructing a clear research question: What is the optimal methodology to assess plasma cell counts in biopsies of patients with CVID-E and what are the quantitative characteristics of plasma cells in the GI-tract of patients with CVID-E? The key concepts that were focused on were the histopathological description, plasma cell quantification in CVID-E and locations described, number of biopsies and sections examined, and classes of plasma cells as found in CVID-E. Further description of the search strategy can be found in the Supplementary materials 1, Supplemental Digital Content, document, the search strings used can be found in Supplementary Table 1, Supplemental Digital Content, and the inclusion/exclusion process is highlighted using a PRISMA diagram in Supplementary Diagram 1, Supplemental Digital Content. Lastly, we performed a risk of bias analysis according to the Cochrane Risk of Bias In Nonrandomized Studies – of Exposures (ROBINS-E) methodology, which can be found in Supplementary materials 2, Supplemental Digital Content, [22]. A plot of this was generated using the robvis tool also provided by the MRC hubs for trials methodology research (Supplementary Figure 1, Supplemental Digital Content) [23].

## RESULTS

### The number of biopsies required for the plasma cell count and the threshold to define paucity of plasma cell count varies between studies

Quantification of plasma cell count, and the number and location of sampling vary between studies. The quantification methods are summarized in Table 1, and the number and locations in Supplementary Table 2, Supplemental Digital Content, [24,25^▪^,^26^,27^▪^]. The proportion of plasma cells in the intestinal lamina propria was determined, semi-quantitatively, in conventional histological preparations stained with haematoxylin and eosin. One of the quantitative measures used is the proportion of plasma cells to other mononuclear cells in CVID in comparison to healthy controls, with a paucity being defined as 1–5% of mononuclear cells. Two other methodologies that closely align to each other are those of Emerson *et al.* and Malamut *et al.*, where ≤10 plasma cells as an average over multiple HPFs is considered a paucity [25^▪^,27^▪^]. Another definition is used by Gullo *et al.*, which considers ≤40 plasma cells at 20× a paucity as less conservative compared to the other methods presented.

**Table 1:** Quantification of plasma cell content in CVID-E biopsies per study

Study:	Quantification:
Herbst *et al.*[22]	Plasma cells in proportion to other mononuclear cells in comparison to controls: Absent: 0% Extremely rare: <1% Decreased: 1–5% Normal: 5–30% Increased: >30%
Malamut *et al.*[25^▪^]	In 10 HPF at magnification 40×, per class: Absent (0): 0 +: 0–10 ++: 10–50 +++: >50 IgA control = +++ IgM control = ++ IgG control = +
Emerson *et al.*[23]	Counts using 3 HPF sections at 40× in the lamina propria: - Absent: none present (after 3 HPF examinations) - Reduced: <10 cells on average per HPF - Normal: >10 cells on average per HPF
Gullo *et al.*[24]	Counts per 20× power field: - Paucity (≤40) - Absent

Left column lists studies included and the right column sets out the methodology as defined by the authors for characterizing plasma cell content in the GI tract. CVID-E, common variable immunodeficiency enteropathy; Ig, immunoglobulin.

According to some authors, plasma cell counts vary with the location of the biopsy, with a significant variation between the colon and duodenum within patients [18^▪^,^28^], whereas other authors report little variation between location of biopsy, with patients exhibiting a consistent reduction in plasma cell count over the entire GI tract [13,29]. Most studies found comparable results on colon biopsies, with paucity or absence of plasma cells noted in 60–85% of patients, with Khan *et al.* being the only exception. All studies, except for Gullo *et al.* and Khan *et al.*, performed duodenal plasma cell analyses. Most studies have analysed multiple biopsies from various locations throughout the GI tract. The locations of these biopsies have been documented in Supplementary Table 2, Supplemental Digital Content. [19^▪▪^,^24^,25^▪^].

Apart from the location of biopsies and method of quantification, the number of biopsies and sections per biopsy required to prevent sampling error in the assessment of plasma cell counts varied. Pehlivanoğlu *et al.* showed that a follow-up biopsy exhibited divergent results in 44% of patients undergoing recurrent biopsies [28], implying that single biopsies are insufficient for the assessment of plasma cell counts. Emerson *et al.* present a methodology with average measures on 3 sections [25^▪^], reducing the chance of sampling error within single biopsies. The number of biopsies taken varies per study and is not standardized.

### Reduced plasma cell counts are found over the entire gastrointestinal-tract but are most reduced in the small intestine

The plasma cell counts over the whole GI tract were compared in Supplementary Table 3, Supplemental Digital Content. We excluded studies not reporting thresholds of plasma cell counts from this analysis. Overall, a paucity or reduction of plasma cells is found. However, the degree of paucity or reduction in biopsies varied heavily between studies, likely due to the variation in assessment method. Furthermore, some studies chose to report results as based on findings on biopsy level while others report results as based on findings on patient level.

We stratified the studies by location in Table 2 to determine whether counts of plasma cells in biopsies differed over the GI-tract. In three of four studies on stomach biopsies, most patients had reduced plasma cell counts. All studies reported a paucity or absence in the small intestine, and only a single study reported predominantly normal plasma cell counts in the colon. Yet, Jørgensen *et al.* and Strohmeier *et al.* reported reduced counts of plasma cells only in a subset of their symptomatic patient cohorts [18^▪^,^19^^▪▪^]. Only Pehlivanoğlu *et al.* measured plasma cells in the oesophagus but found no abnormalities [28]. In conclusion, a paucity or absence of plasma cells was most often found in the small intestine, and was frequently found in the colon and stomach, except by Jørgensen *et al.* and Khan *et al.*[18^▪^,^29^]. Jørgenson *et al.* found predominantly normal plasma cell counts in the stomach, and Khan *et al.* predominantly normal plasma cell counts in the colon. However, there is strong inter-study variability in the degree of paucity and absence and severity of enteropathy symptoms.

**Table 2:** Description of plasma cell paucity or absence in biopsies with CVID as stratified by location

	Location											
	Oesophagus *N* = 5			Gastric *N* = 187			Small intestine *N* = 375			Colon *N* = 168		
Study	Absence *N* = 0	Paucity	Normal *N* = 5 (100%)	Absence *N* = 82 (44%)	Paucity *N* = 29 (16%)	Normal *N* = 76 (40%)	Absence *N* = 168 (45%)	Paucity *N* = 75 (20%)	Normal *N* = 132 (35%)	Absence *N* = 32 (19%)	Paucity *N* = 65 (39%)	Normal *N* = 70 (42%)
Pehlivanoğlu *et al.*^a^[26]	0	–	5	67	–	15	45	–	20	11	–	2
Van Schewick *et al.*^a^[17^▪^]	–	–	–	–	–	–	39	6	9	19	6	4
Jørgensen *et al.*^a,b^[18^▪^]	–	–	–	–	7	43	–	22	30	–	29	23
Washington *et al.*^a^[28]	–	–	–	8	–	1	17	–	0	–	–	–
Khan *et al.*^c^[27^▪^]	–	–	–	–	–	–	–	–	–	–	6	29
Gullo *et al.*[24]	–	–	–	7	2	0	–	–	–	–	–	–
Lougaris *et al.*^a,d^[16]	–	–	–	–	–	–	15	–	6	2	–	1
Emerson *et al.*[23]	–	–	–	–	–	–	8	13	14	–	–	–
Strohmeier *et al.*[19^▪▪^]	–	–	–	–	–	–	25	–	33	–	–	–
Biagi *et al.*[30]	–	–	–	–	–	–	15	–	2	–	–	–
Herbst *et al.*[22]	–	–	–	–	–	–	4	9	4	–	–	–
Daniels *et al.*[13]	–	–	–	–	19	17	–	25	14	–	24	11

Small intestine includes both duodenum, jejunum, and ileum. Colon includes all sections including caecum, ascending, transverse, descending, sigmoid, and rectum. – Indicates that study did not measure plasma cells in this location or did not use this metric for determination plasma cell contents. CVID, common variable immunodeficiency.

a Indicates that studies have metachronous biopsies taken and so multiple biopsies were taken of the same patient.

b Results on duodenal and ileal biopsies are combined into one metric, both sites had similar plasma cell concentration.

c Study did not specify where biopsies from esophagoduodenoscopy were retrieved from, and thus are not included in this table.

d Plasma cell content was not assessed in oesophageal biopsies. *N*= number of biopsies.

### There is a pronounced reduction in immunoglobulin A+ plasma cells in common variable immunodeficiency enteropathy in the gastrointestinal tract

Three studies reported on counts of subsequent plasma cell classes in CVID, as presented in Table 3. These studies show plasma cell reductions in all classes, predominantly IgA^+^ plasma cells in CVID-E patients [19^▪▪^,^24^,27^▪^]. IgM^+^ plasma cell counts were more conserved in the gut compared to IgA^+^ and IgG^+^ plasma cell counts in 2 out of 3 studies [24,19^▪▪^]. Additionally, IgG^+^ plasma cell counts were reduced in all studies, but to a lesser degree compared to IgA^+^. Strohmeier *et al.* found that IgA plasma cell counts were reduced or absent in duodenal samples and absent in all samples with documented villous atrophy (*n* = 20/65) [19^▪▪^]. Herbst *et al.* found that defects in IgA^+^ plasma cell counts correlated with a reduced total plasma cell count in the duodenum [24], and Emerson et. al. found that low plasma cell counts in the duodenum corresponded to low serum IgA levels. Emerson *et al.* however, did not stratify by class and thus their study was not included in Table 3[25^▪^]. Shulzhenko *et al.* reported that CVID-E was more frequent in CVID patients with low IgA plasma cell counts in duodenal tissues compared to patients with normal IgA plasma cell counts [20].

**Table 3:** Description of plasma cell class in studies that examined this feature

	Plasma cell class presence			
Study	IgG^+^*N* = 34/91 (37%)	IgA^+^*N* = 20/87 (23%)	IgM^+^*N* = 38/87 (44%)	Total patients *N* = 93 (100%)
Herbst *et al.*^a^[22]	10/17 (59%)	4/17 (24%)	12/17 (71%)	17
Malamut *et al.*[25^▪^]	2/11 (18%)	3/11 (27%)	2/11 (18%)	11
Strohmeier *et al.*^b^[19^▪▪^]	22/63 (35%)	13/59 (22%)	24/59 (41%)	65

Studies are noted on the first column, whereas plasma cell class and total patients are on following columns. *N*= number of biopsies with presence of plasma cell class examined. Ig, immunoglobulin.

a IgM measurements were stratified by absence, paucity, normal. IgM in this table includes paucity and absence for this study. This study only examined IgG2 due to insufficient staining methodology for IgG1 and unmeasurable IgG3–4 in patients and controls.

b Plasma cell class data was not available for all patients; hence totals do not equal total patients.

## DISCUSSION

### Our proposition for an optimized methodology

Overall, consensus on how to quantify the plasma cell count in the gut is lacking, and the inconsistent methodology used, and varying number of biopsies and sections examined per patient further illustrates this. For practical usage in histopathological assessment, and based on the literature, we recommend an optimized methodology of quantification using a cut-off value of <10 plasma cells per HPF at 40x magnification, or a proportion of ≥1–5% of total mononuclear cells, recorded over ≥3 sections, and in ≥2 biopsies. These are the most conservative agreeable methods which best account for sampling error within patients. In addition, IgA plasma cell counts may be a more accurate predictor for CVID-E. However, several issues remain unresolved including the definition of CVID-E and severity of clinical symptoms.

### Risk of bias and heterogeneity in the literature highlights the need for standardization

Our ROBINS-E analysis shows that across all literature assessing the link between GI plasma cells and CVID there is risk of bias. Although confounding was not an issue, as having absent plasma cells is pathognomonic to CVID, there are inconsistencies in plasma cell assessment that led to potential bias in multiple studies. We believe that a clear consensus on the histopathology is essential to reduce future bias stemming from these inconsistent assessments. All studies showed risk of selection bias, as there are no prospective cohorts including patients without CVID or CVID-E, leading to more studies mostly including CVID patients that required a GI biopsy. This highlights the need for a balanced prospective, or large retrospective cohort. The lack of bias due to interventions was an expected outcome, as plasma cell paucity is not expected to change with any preestablished treatments for CVID or CVID-E. Finally, due to the observational nature of most studies, there was often not a preconceived analysis plan available nor consistent data regarding the number of biopsies assessed per patient. Future studies should ensure that equal numbers of biopsies are assessed per patient, and that biopsies are taken from similar locations according to a preconceived study protocol.

During the documentation of the study populations and study types it became clear that most studies do not define CVID-E in a similar manner. Where 10 of the analysed studies include all CVID patients with endoscopic biopsies [13,16,17^▪^,18^▪^,^24^,25^▪^,^26^,^28^–^30^], 4 other studies explicitly include only patients with CVID and GI symptoms [19^▪▪^,27^▪^,^31^,^32^]. Within this last group, sort and severity of GI symptoms are not consistently defined, resulting in highly heterogenous study populations. Although this partly reflects the heterogenous nature of CVID-E and CVID in general, it is essential for future research that a clear consensus on diagnosis of CVID-E based on present GI symptoms, severity of GI symptoms, and histopathology is reached by the CVID research community. By focusing on paucity of plasma cell counts in this report, we contribute to the histopathological aspect.

### Reduced immunoglobulin A plasma cells and the microbiome might play an important role in mucosal integrity

The relevance of reduced IgA plasma cell counts, and low serum IgA levels indicative of CVID-E needs further support. Yet, current studies on immunopathology, may support including this measure. Shulzhenko *et al.* found that multiple genes were upregulated associated with inhibition of lipid metabolism in CVID-E epithelial cells in a small cohort. In an *in vitro* macrophage co-culture model, they showed that this was mediated through increased expression of interferon type I and type II signatures by macrophages in the presence of *A. baumannii*. The presence of this pathobiont was associated with CVID-E and had a strong negative correlation with low IgA gene expression [20]. Furthermore, Strohmeier *et al.* found that patients with absence of IgA plasma cells displayed a stronger upregulation in genes involved in T-cell activation and cytokine production. IFN type I/III and II response pathways were highly enriched in CVID-E, suggesting a role for increased interferon signalling [19^▪▪^]. This may suggest that increased interferon production through dysbiosis of microbiota could drive changes in epithelial cell lipid metabolism and lead to disease manifestation such as CVID-E.

**FIGURE 1:**
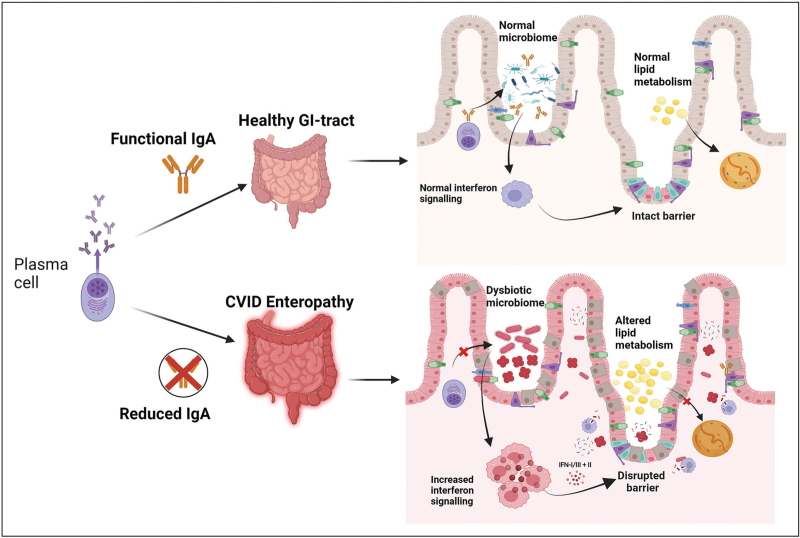
Presumed schematic mechanism of barrier disruption in patients with CVID-E as mediated through IgA deficiency. Top part of the figure shows a healthy gut-epithelial barrier, whereas the bottom shows a disrupted gut-epithelial barrier with a disrupted microbiome. Figure created with BioRender (biorender.com). CVID-E, common variable immunodeficiency enteropathy; IgA, immunoglobulin A.

The full relationship between IgA plasma cell count and severity of GI-symptoms is still unclear, but low serum IgA predisposes for GI infections in a large cohort of 252 CVID patients [33]. In a CD19^-/-^ mouse model it has been shown that antibody deficiency was associated with defective anticommensal IgA responses [34]. Altogether, this suggests an important role for IgA within the integrity of the mucosal tissue in the GI tract. This hypothetical process is visualized in Fig. 1.

### A clear link between total plasma cell counts and gastrointestinal symptoms has not been shown yet

A link between total plasma cell counts and GI symptoms in CVID was not found by Jørgensen *et al.*[18^▪^]. However, patients were not stratified by severity of GI symptoms. Therefore, differences in plasma cell counts between patients with severe vs. mild GI symptoms might be present. Alternatively, subsets of CVID patients not suffering from enteropathy might still have IgA functionality in their gut mucosa, or subsets of patients within CVID have different clinical manifestations based on underlying characteristics beyond plasma cell count and class. These findings highlight the importance of stratification by symptom between CVID phenotypes and that further systematic investigations are needed in CVID-E.

## CONCLUSION

In conclusion, adequate assessment of plasma cell count should be done through quantification using a cut-off value of <10 plasma cells per HPF at 40× magnification, or a proportion of ≤1–5% of total mononuclear cells, recorded over ≥3 sections, and in ≥2 biopsies. These approaches are expected to yield the most accurate results [25^▪^,27^▪^,^30^]. The optimal location of assessment according to our findings is the small intestine, as all studies found a paucity in this location. That being said, most studies also reported a paucity in the stomach and colon. In all studies, a reduction in plasma cells was reported to be associated with CVID, confirming the hypothesis that this is a significant histological feature of this patient subtype. However, a subset of patients suffers from GI symptoms with no histological plasma cell alterations. Whether the severity of these symptoms would indicate CVID-E remains unresolved. We showed that reduced plasma cell counts can be found over the entire GI-tract except for the oesophagus, but there is strong inter-study variability in the distribution of paucity and absence compared to normal plasma cell counts. Finally, CVID-E patients seem to have lower counts of IgA+ plasma cells in the GI-tract than other CVID patients. Although potentially promising, future studies are needed for confirmation in larger cohorts to elucidate the role of reductions in IgA+ plasma cells in CVID-E [19^▪▪^,^24^,27^▪^].

## Acknowledgements

*Figure 1* *was created in BioRender (biorender.com).*

*Author contributions: J.W.N.M. and H.L.L. designed and conceived the study. The search string and search strategy was constructed by J.W.N.M., with feedback from H.L.L. Feedback and input on pathological assessment was provided by M.M.L., and M.S. provided feedback and input on the content of the manuscript.*

*Supplementary materials: Further information on the construction of the search string and inclusion/exclusion process can be found in the supplementary materials 1*. *These include a small text description of the construction of the search string, a table with the key terms, synonyms, and the search strings used, and a PRISM diagram detailing the inclusion/exclusion process. Supplementary material 2, contains our results of our ROBINS-E analysis with a complementary stoplight plot.*

*Inclusion and diversity: We support inclusive, diverse, and equitable conduct of research.*

### Financial support and sponsorship

*None.*

### Conflicts of interest

*The authors of this manuscript certify that they have no affiliations with any organizations or entities that have any financial or nonfinancial interest in the subject matter or materials discussed in this manuscript. The authors have declared no competing interest.*

## Supplementary Material

## REFERENCES AND RECOMMENDED READING

Papers of particular interest, published within the annual period of review, have been highlighted as:

▪ of special interest
▪▪ of outstanding interest
