# Supplementary material for: Paucity of gastrointestinal plasma cells in common variable immunodeficiency
Source: Curr Opin Allergy Clin Immunol. 2024 Oct 7;24(6):464–71. doi: 10.1097/ACI.0000000000001040 (PMC11537466; doi:10.1097/ACI.0000000000001040)
Supplement: Supplementary file 1 [file coaci-24-464-s001.docx]

**Supplementary materials 1:**

We used a SPICE (setting, perspective, intervention/exposure/interest, comparison, and evaluation) format for assessment of qualitative features of a disease.

A search string was constructed based on the following key words: Common Variable Immuno-deficiency, enteropathy, histopathology, plasma cells, intra-epithelial lymphocyte, inflammatory bowel disease, gastritis and gastropathy, metaplasia, intestinal villous atrophy, colitis, inflammation, lymphangiectasis, lymphoid hyperplasia, and graft vs host disease. All features have been associated with the histopathological picture ^13^ or serve as disease mimics ^10, 18^.

Terms were chosen based on the thesaurus of the EMBASE and PubMed databases and were either index terms of EMBASE and PUBMED trees or related synonyms. The search terms all included variations, which are documented together with the search string and key terms used in supplementary materials Table 1. Within the search string itself, we ensured that abbreviations were included in the terms used and that it was constructed with extensive feedback from experienced clinical immunologists. We ensured that the search was optimized and all errors in the string were removed prior to translation of the search string to other databases

The inclusion and exclusion processes are described in Supplementary Diagram 1. We initially removed duplicate records before screening them on abstract and title. The exclusion criteria were constructed so that that the disease topic was CVID, the papers assessed plasma cell content in the GI tract in a quantitative or qualitative manner, and that no reviews, posters, meta-analysis, or outdated papers were included. Only papers in English were included. The required features of plasma cells for inclusion were one or a combination of: a noted increase or decrease, location of plasma cell deficiency, plasma cell quality, and number of sections or biopsies assessed for plasma cell evaluation.

*Supplementary Table 1: Key terms, synonyms of said key terms, and the search strings used to examine the databases of EMBASE and PUBMED with the amount of unique hits included. The search string was initially constructed in EMBASE, and afterwards translated to PUBMED.*

| Key terms: | Common variable immuno-deficiency, CVID,  Enteropathy, gut inflammation, gastrointestinal inflammation,  Treatment  Histopathology  Plasma Cells  Intra-epithelial lymphocyte  Inflammatory bowel disease, IBD  Gastritis and gastropathy  Metaplasia  Intestinal villous atrophy  Colitis  Inflammation  Lymphangiectasis  Lymphoid hyperplasia  Graft vs host disease |
| --- | --- |
| Synonyms: | Common variable immune deficiency  Enteropathy  Treatment – Treatment, Therapy  Histopathology - microscopy  Plasma Cells – Plasma cell, absence of plasma cell  Intra-epithelial lymphocyte - IEL  Inflammatory bowel disease – crypt distortion, granulomatous disease  Gastritis and gastropathy – Gastritis and Stomach disease, atrophic gastritis  Metaplasia – gastric carcinoma  Intestinal villous atrophy – Intestine villous atrophy, villous blunting  Colitis – Enteritis, colitis.  Inflammation  Lymphangiectasis – intestine lymphangiectasia, lymphangiectasia  Lymphoid hyperplasia – Follicular lymphoid hyperplasia, lymphoid hyperplasia, lymphoid aggregates, granulomas, nodular lymphoid hyperplasia  Graft vs host disease – GvHD |
| Search string EMBASE (2.405 hits): | (('common variable immunodeficiency'/exp OR 'common variable immunodeficiency') AND ('enteropathy'/exp OR 'enteropathy' OR 'gut inflammation' OR 'gastrointestinal inflammation') AND ('histology'/exp OR 'histology' OR 'histopathology'/exp OR 'histopathology' OR 'microscopy' OR 'microscopic') OR (('common variable immunodeficiency'/exp OR 'common variable immunodeficiency') AND ('enteropathy'/exp OR 'enteropathy' OR 'gut inflammation' OR 'gastrointestinal inflammation') AND ('therapy'/exp OR 'therapy' OR 'treatment'/exp OR 'treatment')) OR (('common variable immunodeficiency'/exp OR 'common variable immunodeficiency') AND ('enteropathy'/exp OR 'enteropathy' OR 'gut inflammation' OR 'gastrointestinal inflammation') AND ('therapy'/exp OR 'therapy' OR 'treatment'/exp OR 'treatment') AND ('histology'/exp OR 'histology' OR 'histopathology'/exp OR 'histopathology' OR 'microscopy')) OR (('common variable immunodeficiency'/exp OR 'common variable immunodeficiency') AND ('enteropathy'/exp OR 'enteropathy' OR 'gut inflammation' OR 'gastrointestinal inflammation') AND ('plasma cell'/exp OR 'plasma cell' OR 'absence of plasma cell') AND ('gastritis'/exp OR 'gastritis' OR 'stomach disease' OR 'gastropathy' OR 'atrophic gastritis'/exp OR 'atrophic gastritis') AND ('inflammatory bowel disease'/exp OR 'inflammatory bowel disease' OR 'crypt distortion' OR 'granulomatous inflammation'/exp OR 'granulomatous inflammation') AND ('intraepithelial lymphocyte'/exp OR 'intraepithelial lymphocyte' OR 'iel' OR 'sub-endothelial lymphocytosis') AND ('metaplasia'/exp OR 'metaplasia') AND ('intestine villous atrophy' OR 'villous atrophy' OR 'villous blunting') AND ('enteritis'/exp OR 'enteritis' OR 'gastritis'/exp OR 'gastritis') AND ('inflammation'/exp OR 'inflammation') AND ('intestine lymphangiectasia'/exp OR 'intestine lymphangiectasia') AND ('graft versus host reaction'/exp OR 'gvhd' OR 'graft versus host disease' OR 'graft versus host reaction')) OR (('common variable immunodeficiency'/exp OR 'common variable immunodeficiency') AND ('plasma cell'/exp OR 'plasma cell' OR 'absence of plasma cell')) OR (('common variable immunodeficiency'/exp OR 'common variable immunodeficiency') AND ('lymphoid hyperplasia'/exp OR 'lymphoid hyperplasia' OR 'follicular lymphoid hyperplasia' OR 'lymphoid aggregates' OR 'granuloma' OR 'nodular lymphoid hyperplasia' OR 'follicular lymphoma'/exp)) OR (('common variable immunodeficiency'/exp OR 'common variable immunodeficiency') AND ('graft versus host reaction'/exp OR 'gvhd' OR 'graft versus host disease' OR 'graft versus host reaction')) OR (('common variable immunodeficiency'/exp OR 'common variable immunodeficiency') AND ('inflammatory bowel disease'/exp OR 'inflammatory bowel disease' OR 'crypt distortion' OR 'granulomatous inflammation'/exp OR 'granulomatous inflammation')) OR (('common variable immunodeficiency'/exp OR 'common variable immunodeficiency') AND ('enteropathy'/exp OR 'enteropathy' OR 'gut inflammation' OR 'gastrointestinal inflammation') AND ('intestine villous atrophy' OR 'villous atrophy' OR 'villous blunting')) OR (('common variable immunodeficiency'/exp OR 'common variable immunodeficiency') AND ('enteropathy'/exp OR 'enteropathy' OR 'gut inflammation' OR 'gastrointestinal inflammation') AND ('plasma cell'/exp OR 'plasma cell' OR 'absence of plasma cell')) OR (('common variable immunodeficiency'/exp OR 'common variable immunodeficiency') AND ('enteropathy'/exp OR 'enteropathy' OR 'gut inflammation' OR 'gastrointestinal inflammation') AND ('gastritis'/exp OR 'gastritis' OR 'stomach disease' OR 'gastropathy' OR 'atrophic gastritis'/exp OR 'atrophic gastritis')) OR (('common variable immunodeficiency'/exp OR 'common variable immunodeficiency') AND ('enteropathy'/exp OR 'enteropathy' OR 'gut inflammation' OR 'gastrointestinal inflammation') AND ('intraepithelial lymphocyte'/exp OR 'intraepithelial lymphocyte' OR 'iel' OR 'sub-epithelial lymphocytosis')) OR (('common variable immunodeficiency'/exp OR 'common variable immunodeficiency') AND ('enteropathy'/exp OR 'enteropathy' OR 'gut inflammation' OR 'gastrointestinal inflammation') AND ('metaplasia'/exp OR 'metaplasia')) OR (('common variable immunodeficiency'/exp OR 'common variable immunodeficiency') AND ('enteropathy'/exp OR 'enteropathy' OR 'gut inflammation' OR 'gastrointestinal inflammation') AND ('enteritis'/exp OR 'enteritis' OR 'gastritis'/exp OR 'gastritis' OR 'stomach disease')) OR (('common variable immunodeficiency'/exp OR 'common variable immunodeficiency') AND ('enteropathy'/exp OR 'enteropathy' OR 'gut inflammation' OR 'gastrointestinal inflammation') AND ('inflammation'/exp OR 'inflammation')) OR (('common variable immunodeficiency'/exp OR 'common variable immunodeficiency') AND ('enteropathy'/exp OR 'enteropathy' OR 'gut inflammation' OR 'gastrointestinal inflammation') AND ('intestine lymphangiectasia'/exp OR 'intestine lymphangiectasia'))) AND ([embase]/lim OR [medline]/lim OR [pubmed-not-medline]/lim) |
| Search string PUBMED version (457 hits): | (('common variable immunodeficiency' [MESH] OR 'common variable immunodeficiency'[tiab]) AND ('enteropathy'[MESH] OR 'enteropathy'[tiab] OR ‘gut inflammation’[tiab] OR ‘gastrointestinal inflammation’[tiab]) AND ('histology'[MESH] OR 'histology'[tiab] OR 'histopathology'[MESH] OR 'histopathology'[tiab] OR 'microscopy'[tiab] OR 'microscopic'[tiab]) OR (('common variable immunodeficiency'[MESH] OR 'common variable immunodeficiency'[tiab]) AND ('enteropathy'[MESH] OR 'enteropathy'[tiab] OR 'gut inflammation'[tiab] OR 'gastrointestinal inflammation'[tiab]) AND ('therapy'[MESH] OR 'therapy'[tiab] OR 'treatment'[MESH] OR 'treatment'[tiab])) OR (('common variable immunodeficiency'[MESH] OR 'common variable immunodeficiency'[tiab]) AND ('enteropathy'[MESH] OR 'enteropathy'[tiab] OR 'gut inflammation'[tiab] OR 'gastrointestinal inflammation'[tiab]) AND ('therapy'[MESH] OR 'therapy'[tiab] OR 'treatment'[MESH] OR 'treatment'[tiab]) AND ('histology'[MESH] OR 'histology'[tiab] OR 'histopathology'[MESH] OR 'histopathology'[tiab] OR 'microscopy'[tiab])) OR (('common variable immunodeficiency'[MESH] OR 'common variable immunodeficiency'[tiab]) AND ('enteropathy'[MESH] OR 'enteropathy'[tiab] OR 'gut inflammation'[tiab] OR 'gastrointestinal inflammation'[tiab]) AND ('plasma cell'[MESH] OR 'plasma cell'[tiab] OR 'absence of plasma cell'[tiab]) AND ('gastritis'[MESH] OR 'gastritis'[tiab] OR 'stomach disease'[tiab] OR 'gastropathy'[tiab] OR 'atrophic gastritis'[MESH] OR 'atrophic gastritis'[tiab]) AND ('inflammatory bowel disease'[MESH] OR 'inflammatory bowel disease'[tiab] OR 'crypt distortion'[tiab] OR 'granulomatous inflammation'[MESH] OR 'granulomatous inflammation'[tiab]) AND ('intraepithelial lymphocyte’[MESH] OR 'intraepithelial lymphocyte'[tiab] OR 'iel'[tiab] OR 'sub-endothelial lymphocytosis'[tiab]) AND ('metaplasia'[MESH] OR 'metaplasia'[tiab]) AND ('intestine villous atrophy'[tiab] OR 'villous atrophy'[tiab] OR 'villous blunting'[tiab]) AND ('enteritis'[MESH] OR 'enteritis'[tiab] OR 'gastritis'[MESH] OR 'gastritis'[tiab]) AND ('inflammation'[MESH] OR 'inflammation'[tiab]) AND ('intestine lymphangiectasia'[MESH] OR 'intestine lymphangiectasia'[tiab]) AND ('graft versus host reaction'[MESH] OR 'gvhd'[tiab] OR 'graft versus host disease'[tiab] OR 'graft versus host reaction'[tiab])) OR (('common variable immunodeficiency'[MESH] OR 'common variable immunodeficiency'[tiab]) AND ('plasma cell'[MESH] OR 'plasma cell'[tiab] OR 'absence of plasma cell'[tiab])) OR (('common variable immunodeficiency'[MESH] OR 'common variable immunodeficiency'[tiab]) AND ('lymphoid hyperplasia'[MESH] OR 'lymphoid hyperplasia'[tiab] OR 'follicular lymphoid hyperplasia’[tiab] OR 'lymphoid aggregates'[tiab] OR 'granuloma'[tiab] OR 'nodular lymphoid hyperplasia'[tiab] OR 'follicular lymphoma'[MESH])) OR (('common variable immunodeficiency'[MESH] OR 'common variable immunodeficiency'[tiab]) AND ('graft versus host reaction'[MESH] OR 'gvhd'[tiab] OR 'graft versus host disease'[tiab] OR 'graft versus host reaction'[tiab])) OR (('common variable immunodeficiency'[MESH] OR 'common variable immunodeficiency'[tiab]) AND ('inflammatory bowel disease'[MESH] OR 'inflammatory bowel disease'[tiab] OR 'crypt distortion'[tiab] OR 'granulomatous inflammation'[MESH] OR 'granulomatous inflammation'[tiab])) OR (('common variable immunodeficiency'[MESH] OR 'common variable immunodeficiency'[tiab]) AND ('enteropathy'[MESH] OR 'enteropathy'[tiab] OR 'gut inflammation'[tiab] OR 'gastrointestinal inflammation'[tiab]) AND ('intestine villous atrophy'[tiab] OR 'villous atrophy'[tiab] OR 'villous blunting'[tiab])) OR (('common variable immunodeficiency'[MESH] OR 'common variable immunodeficiency'[tiab]) AND ('enteropathy'[MESH] OR 'enteropathy'[tiab] OR 'gut inflammation'[tiab] OR 'gastrointestinal inflammation'[tiab]) AND ('plasma cell'[MESH] OR 'plasma cell' OR 'absence of plasma cell')) OR (('common variable immunodeficiency'[MESH] OR 'common variable immunodeficiency'[tiab]) AND ('enteropathy'[MESH] OR 'enteropathy'[tiab] OR 'gut inflammation'[tiab] OR 'gastrointestinal inflammation'[tiab]) AND ('gastritis'[MESH] OR 'gastritis'[tiab] OR 'stomach disease'[tiab] OR 'gastropathy'[tiab] OR 'atrophic gastritis'[MESH] OR 'atrophic gastritis'[tiab])) OR (('common variable immunodeficiency'[MESH] OR 'common variable immunodeficiency'[tiab]) AND ('enteropathy'[MESH] OR 'enteropathy'[tiab] OR 'gut inflammation'[tiab] OR 'gastrointestinal inflammation'[tiab]) AND ('intraepithelial lymphocyte'[MESH] OR 'intraepithelial lymphocyte'[tiab] OR 'iel'[tiab] OR 'sub-epithelial lymphocytosis'[tiab])) OR (('common variable immunodeficiency'[MESH] OR 'common variable immunodeficiency'[tiab]) AND ('enteropathy'[MESH] OR 'enteropathy'[tiab] OR 'gut inflammation'[tiab] OR 'gastrointestinal inflammation'[tiab]) AND ('metaplasia'[MESH] OR 'metaplasia'[tiab])) OR (('common variable immunodeficiency'[MESH] OR 'common variable immunodeficiency'[tiab]) AND ('enteropathy'[MESH] OR 'enteropathy'[tiab] OR 'gut inflammation'[tiab] OR 'gastrointestinal inflammation'[tiab]) AND ('enteritis'[MESH] OR 'enteritis'[tiab] OR 'gastritis'[MESH] OR 'gastritis'[tiab] OR 'stomach disease'[tiab])) OR (('common variable immunodeficiency'[MESH] OR 'common variable immunodeficiency'[tiab]) AND ('enteropathy'[MESH] OR 'enteropathy'[tiab] OR 'gut inflammation'[tiab] OR 'gastrointestinal inflammation'[tiab]) AND ('inflammation'[MESH] OR 'inflammation'[tiab])) OR (('common variable immunodeficiency'[MESH] OR 'common variable immunodeficiency'[tiab]) AND ('enteropathy’[MESH] OR 'enteropathy'[tiab] OR 'gut inflammation'[tiab] OR 'gastrointestinal inflammation'[tiab]) AND ('intestine lymphangiectasia'[MESH] OR 'intestine lymphangiectasia'[tiab]))) |

*Supplementary Diagram 1: PRISMA diagram detailing search process. The first stage details the identification of duplicate records and databases + registers used in the pre-screening process. The second stage details the abstract and title screening, the global screening, and the full-text screening. Exclusion criteria are described on the right-hand side of the diagram.*

**Identification of studies via databases and registers**

**Screening**

Papers screened on abstract and title.

(n = 2,942)

Papers assessed using global screening.

(n = 74)

**Identification**

Records identified from EMBASE and PubMed:

Databases (n = 2)

Registers (n = 2)

Records removed *before screening*:

Duplicate records removed (n = 491)

Records marked as ineligible by automation tools (n = 0)

Records removed for other reasons (n = 0)

Records excluded

(n = 2,868) and reasons:

- Not related to CVID
- Not related to CVID enteropathy
- No mention of CVID enteropathy histopathology or CVID enteropathy.
- No mention of plasma cells
- Posters
- Primary language was not English or Dutch.
- Review

Excluded papers and reasons(n = 39):

- Paper before 1990
- No qualitative or quantitative description plasma cell content.
- Posters or abstract only.
- Primary language not in English or Dutch.
- No access to full text

Reports assessed for eligibility through full text screening.

(n = 35)

Reports excluded: N = 3:

- Records focused solely on treatment effect.

**Included**

Studies included in review.

(n = 32)
