# Supplementary material for: Paucity of gastrointestinal plasma cells in common variable immunodeficiency
Source: Curr Opin Allergy Clin Immunol. 2024 Oct 7;24(6):464–71. doi: 10.1097/ACI.0000000000001040 (PMC11537466; doi:10.1097/ACI.0000000000001040)
Supplement: Supplementary file 2 [file coaci-24-464-s002.docx]

*Supplementary Table 1: Key terms, synonyms of said key terms, and the search strings used to examine the databases of EMBASE and PUBMED with the amount of unique hits included. The search string was initially constructed in EMBASE, and afterwards translated to PUBMED.*

| Key terms: | Common variable immuno-deficiency, CVID,  Enteropathy, gut inflammation, gastrointestinal inflammation,  Treatment  Histopathology  Plasma Cells  Intra-epithelial lymphocyte  Inflammatory bowel disease, IBD  Gastritis and gastropathy  Metaplasia  Intestinal villous atrophy  Colitis  Inflammation  Lymphangiectasis  Lymphoid hyperplasia  Graft vs host disease |
| --- | --- |
| Synonyms: | Common variable immune deficiency  Enteropathy  Treatment – Treatment, Therapy  Histopathology - microscopy  Plasma Cells – Plasma cell, absence of plasma cell  Intra-epithelial lymphocyte - IEL  Inflammatory bowel disease – crypt distortion, granulomatous disease  Gastritis and gastropathy – Gastritis and Stomach disease, atrophic gastritis  Metaplasia – gastric carcinoma  Intestinal villous atrophy – Intestine villous atrophy, villous blunting  Colitis – Enteritis, colitis.  Inflammation  Lymphangiectasis – intestine lymphangiectasia, lymphangiectasia  Lymphoid hyperplasia – Follicular lymphoid hyperplasia, lymphoid hyperplasia, lymphoid aggregates, granulomas, nodular lymphoid hyperplasia  Graft vs host disease – GvHD |
| Search string EMBASE (2.405 hits): | (('common variable immunodeficiency'/exp OR 'common variable immunodeficiency') AND ('enteropathy'/exp OR 'enteropathy' OR 'gut inflammation' OR 'gastrointestinal inflammation') AND ('histology'/exp OR 'histology' OR 'histopathology'/exp OR 'histopathology' OR 'microscopy' OR 'microscopic') OR (('common variable immunodeficiency'/exp OR 'common variable immunodeficiency') AND ('enteropathy'/exp OR 'enteropathy' OR 'gut inflammation' OR 'gastrointestinal inflammation') AND ('therapy'/exp OR 'therapy' OR 'treatment'/exp OR 'treatment')) OR (('common variable immunodeficiency'/exp OR 'common variable immunodeficiency') AND ('enteropathy'/exp OR 'enteropathy' OR 'gut inflammation' OR 'gastrointestinal inflammation') AND ('therapy'/exp OR 'therapy' OR 'treatment'/exp OR 'treatment') AND ('histology'/exp OR 'histology' OR 'histopathology'/exp OR 'histopathology' OR 'microscopy')) OR (('common variable immunodeficiency'/exp OR 'common variable immunodeficiency') AND ('enteropathy'/exp OR 'enteropathy' OR 'gut inflammation' OR 'gastrointestinal inflammation') AND ('plasma cell'/exp OR 'plasma cell' OR 'absence of plasma cell') AND ('gastritis'/exp OR 'gastritis' OR 'stomach disease' OR 'gastropathy' OR 'atrophic gastritis'/exp OR 'atrophic gastritis') AND ('inflammatory bowel disease'/exp OR 'inflammatory bowel disease' OR 'crypt distortion' OR 'granulomatous inflammation'/exp OR 'granulomatous inflammation') AND ('intraepithelial lymphocyte'/exp OR 'intraepithelial lymphocyte' OR 'iel' OR 'sub-endothelial lymphocytosis') AND ('metaplasia'/exp OR 'metaplasia') AND ('intestine villous atrophy' OR 'villous atrophy' OR 'villous blunting') AND ('enteritis'/exp OR 'enteritis' OR 'gastritis'/exp OR 'gastritis') AND ('inflammation'/exp OR 'inflammation') AND ('intestine lymphangiectasia'/exp OR 'intestine lymphangiectasia') AND ('graft versus host reaction'/exp OR 'gvhd' OR 'graft versus host disease' OR 'graft versus host reaction')) OR (('common variable immunodeficiency'/exp OR 'common variable immunodeficiency') AND ('plasma cell'/exp OR 'plasma cell' OR 'absence of plasma cell')) OR (('common variable immunodeficiency'/exp OR 'common variable immunodeficiency') AND ('lymphoid hyperplasia'/exp OR 'lymphoid hyperplasia' OR 'follicular lymphoid hyperplasia' OR 'lymphoid aggregates' OR 'granuloma' OR 'nodular lymphoid hyperplasia' OR 'follicular lymphoma'/exp)) OR (('common variable immunodeficiency'/exp OR 'common variable immunodeficiency') AND ('graft versus host reaction'/exp OR 'gvhd' OR 'graft versus host disease' OR 'graft versus host reaction')) OR (('common variable immunodeficiency'/exp OR 'common variable immunodeficiency') AND ('inflammatory bowel disease'/exp OR 'inflammatory bowel disease' OR 'crypt distortion' OR 'granulomatous inflammation'/exp OR 'granulomatous inflammation')) OR (('common variable immunodeficiency'/exp OR 'common variable immunodeficiency') AND ('enteropathy'/exp OR 'enteropathy' OR 'gut inflammation' OR 'gastrointestinal inflammation') AND ('intestine villous atrophy' OR 'villous atrophy' OR 'villous blunting')) OR (('common variable immunodeficiency'/exp OR 'common variable immunodeficiency') AND ('enteropathy'/exp OR 'enteropathy' OR 'gut inflammation' OR 'gastrointestinal inflammation') AND ('plasma cell'/exp OR 'plasma cell' OR 'absence of plasma cell')) OR (('common variable immunodeficiency'/exp OR 'common variable immunodeficiency') AND ('enteropathy'/exp OR 'enteropathy' OR 'gut inflammation' OR 'gastrointestinal inflammation') AND ('gastritis'/exp OR 'gastritis' OR 'stomach disease' OR 'gastropathy' OR 'atrophic gastritis'/exp OR 'atrophic gastritis')) OR (('common variable immunodeficiency'/exp OR 'common variable immunodeficiency') AND ('enteropathy'/exp OR 'enteropathy' OR 'gut inflammation' OR 'gastrointestinal inflammation') AND ('intraepithelial lymphocyte'/exp OR 'intraepithelial lymphocyte' OR 'iel' OR 'sub-epithelial lymphocytosis')) OR (('common variable immunodeficiency'/exp OR 'common variable immunodeficiency') AND ('enteropathy'/exp OR 'enteropathy' OR 'gut inflammation' OR 'gastrointestinal inflammation') AND ('metaplasia'/exp OR 'metaplasia')) OR (('common variable immunodeficiency'/exp OR 'common variable immunodeficiency') AND ('enteropathy'/exp OR 'enteropathy' OR 'gut inflammation' OR 'gastrointestinal inflammation') AND ('enteritis'/exp OR 'enteritis' OR 'gastritis'/exp OR 'gastritis' OR 'stomach disease')) OR (('common variable immunodeficiency'/exp OR 'common variable immunodeficiency') AND ('enteropathy'/exp OR 'enteropathy' OR 'gut inflammation' OR 'gastrointestinal inflammation') AND ('inflammation'/exp OR 'inflammation')) OR (('common variable immunodeficiency'/exp OR 'common variable immunodeficiency') AND ('enteropathy'/exp OR 'enteropathy' OR 'gut inflammation' OR 'gastrointestinal inflammation') AND ('intestine lymphangiectasia'/exp OR 'intestine lymphangiectasia'))) AND ([embase]/lim OR [medline]/lim OR [pubmed-not-medline]/lim) |
| Search string PUBMED version (457 hits): | (('common variable immunodeficiency' [MESH] OR 'common variable immunodeficiency'[tiab]) AND ('enteropathy'[MESH] OR 'enteropathy'[tiab] OR ‘gut inflammation’[tiab] OR ‘gastrointestinal inflammation’[tiab]) AND ('histology'[MESH] OR 'histology'[tiab] OR 'histopathology'[MESH] OR 'histopathology'[tiab] OR 'microscopy'[tiab] OR 'microscopic'[tiab]) OR (('common variable immunodeficiency'[MESH] OR 'common variable immunodeficiency'[tiab]) AND ('enteropathy'[MESH] OR 'enteropathy'[tiab] OR 'gut inflammation'[tiab] OR 'gastrointestinal inflammation'[tiab]) AND ('therapy'[MESH] OR 'therapy'[tiab] OR 'treatment'[MESH] OR 'treatment'[tiab])) OR (('common variable immunodeficiency'[MESH] OR 'common variable immunodeficiency'[tiab]) AND ('enteropathy'[MESH] OR 'enteropathy'[tiab] OR 'gut inflammation'[tiab] OR 'gastrointestinal inflammation'[tiab]) AND ('therapy'[MESH] OR 'therapy'[tiab] OR 'treatment'[MESH] OR 'treatment'[tiab]) AND ('histology'[MESH] OR 'histology'[tiab] OR 'histopathology'[MESH] OR 'histopathology'[tiab] OR 'microscopy'[tiab])) OR (('common variable immunodeficiency'[MESH] OR 'common variable immunodeficiency'[tiab]) AND ('enteropathy'[MESH] OR 'enteropathy'[tiab] OR 'gut inflammation'[tiab] OR 'gastrointestinal inflammation'[tiab]) AND ('plasma cell'[MESH] OR 'plasma cell'[tiab] OR 'absence of plasma cell'[tiab]) AND ('gastritis'[MESH] OR 'gastritis'[tiab] OR 'stomach disease'[tiab] OR 'gastropathy'[tiab] OR 'atrophic gastritis'[MESH] OR 'atrophic gastritis'[tiab]) AND ('inflammatory bowel disease'[MESH] OR 'inflammatory bowel disease'[tiab] OR 'crypt distortion'[tiab] OR 'granulomatous inflammation'[MESH] OR 'granulomatous inflammation'[tiab]) AND ('intraepithelial lymphocyte’[MESH] OR 'intraepithelial lymphocyte'[tiab] OR 'iel'[tiab] OR 'sub-endothelial lymphocytosis'[tiab]) AND ('metaplasia'[MESH] OR 'metaplasia'[tiab]) AND ('intestine villous atrophy'[tiab] OR 'villous atrophy'[tiab] OR 'villous blunting'[tiab]) AND ('enteritis'[MESH] OR 'enteritis'[tiab] OR 'gastritis'[MESH] OR 'gastritis'[tiab]) AND ('inflammation'[MESH] OR 'inflammation'[tiab]) AND ('intestine lymphangiectasia'[MESH] OR 'intestine lymphangiectasia'[tiab]) AND ('graft versus host reaction'[MESH] OR 'gvhd'[tiab] OR 'graft versus host disease'[tiab] OR 'graft versus host reaction'[tiab])) OR (('common variable immunodeficiency'[MESH] OR 'common variable immunodeficiency'[tiab]) AND ('plasma cell'[MESH] OR 'plasma cell'[tiab] OR 'absence of plasma cell'[tiab])) OR (('common variable immunodeficiency'[MESH] OR 'common variable immunodeficiency'[tiab]) AND ('lymphoid hyperplasia'[MESH] OR 'lymphoid hyperplasia'[tiab] OR 'follicular lymphoid hyperplasia’[tiab] OR 'lymphoid aggregates'[tiab] OR 'granuloma'[tiab] OR 'nodular lymphoid hyperplasia'[tiab] OR 'follicular lymphoma'[MESH])) OR (('common variable immunodeficiency'[MESH] OR 'common variable immunodeficiency'[tiab]) AND ('graft versus host reaction'[MESH] OR 'gvhd'[tiab] OR 'graft versus host disease'[tiab] OR 'graft versus host reaction'[tiab])) OR (('common variable immunodeficiency'[MESH] OR 'common variable immunodeficiency'[tiab]) AND ('inflammatory bowel disease'[MESH] OR 'inflammatory bowel disease'[tiab] OR 'crypt distortion'[tiab] OR 'granulomatous inflammation'[MESH] OR 'granulomatous inflammation'[tiab])) OR (('common variable immunodeficiency'[MESH] OR 'common variable immunodeficiency'[tiab]) AND ('enteropathy'[MESH] OR 'enteropathy'[tiab] OR 'gut inflammation'[tiab] OR 'gastrointestinal inflammation'[tiab]) AND ('intestine villous atrophy'[tiab] OR 'villous atrophy'[tiab] OR 'villous blunting'[tiab])) OR (('common variable immunodeficiency'[MESH] OR 'common variable immunodeficiency'[tiab]) AND ('enteropathy'[MESH] OR 'enteropathy'[tiab] OR 'gut inflammation'[tiab] OR 'gastrointestinal inflammation'[tiab]) AND ('plasma cell'[MESH] OR 'plasma cell' OR 'absence of plasma cell')) OR (('common variable immunodeficiency'[MESH] OR 'common variable immunodeficiency'[tiab]) AND ('enteropathy'[MESH] OR 'enteropathy'[tiab] OR 'gut inflammation'[tiab] OR 'gastrointestinal inflammation'[tiab]) AND ('gastritis'[MESH] OR 'gastritis'[tiab] OR 'stomach disease'[tiab] OR 'gastropathy'[tiab] OR 'atrophic gastritis'[MESH] OR 'atrophic gastritis'[tiab])) OR (('common variable immunodeficiency'[MESH] OR 'common variable immunodeficiency'[tiab]) AND ('enteropathy'[MESH] OR 'enteropathy'[tiab] OR 'gut inflammation'[tiab] OR 'gastrointestinal inflammation'[tiab]) AND ('intraepithelial lymphocyte'[MESH] OR 'intraepithelial lymphocyte'[tiab] OR 'iel'[tiab] OR 'sub-epithelial lymphocytosis'[tiab])) OR (('common variable immunodeficiency'[MESH] OR 'common variable immunodeficiency'[tiab]) AND ('enteropathy'[MESH] OR 'enteropathy'[tiab] OR 'gut inflammation'[tiab] OR 'gastrointestinal inflammation'[tiab]) AND ('metaplasia'[MESH] OR 'metaplasia'[tiab])) OR (('common variable immunodeficiency'[MESH] OR 'common variable immunodeficiency'[tiab]) AND ('enteropathy'[MESH] OR 'enteropathy'[tiab] OR 'gut inflammation'[tiab] OR 'gastrointestinal inflammation'[tiab]) AND ('enteritis'[MESH] OR 'enteritis'[tiab] OR 'gastritis'[MESH] OR 'gastritis'[tiab] OR 'stomach disease'[tiab])) OR (('common variable immunodeficiency'[MESH] OR 'common variable immunodeficiency'[tiab]) AND ('enteropathy'[MESH] OR 'enteropathy'[tiab] OR 'gut inflammation'[tiab] OR 'gastrointestinal inflammation'[tiab]) AND ('inflammation'[MESH] OR 'inflammation'[tiab])) OR (('common variable immunodeficiency'[MESH] OR 'common variable immunodeficiency'[tiab]) AND ('enteropathy’[MESH] OR 'enteropathy'[tiab] OR 'gut inflammation'[tiab] OR 'gastrointestinal inflammation'[tiab]) AND ('intestine lymphangiectasia'[MESH] OR 'intestine lymphangiectasia'[tiab]))) |
