# Supplementary material for: Paucity of gastrointestinal plasma cells in common variable immunodeficiency
Source: Curr Opin Allergy Clin Immunol. 2024 Oct 7;24(6):464–71. doi: 10.1097/ACI.0000000000001040 (PMC11537466; doi:10.1097/ACI.0000000000001040)
Supplement: Supplementary file 3 [file coaci-24-464-s003.docx]

*Supplementary Diagram 1: PRISMA diagram detailing search process. The first stage details the identification of duplicate records and databases + registers used in the pre-screening process. The second stage details the abstract and title screening, the global screening, and the full-text screening. Exclusion criteria are described on the right-hand side of the diagram.*

**Identification of studies via databases and registers**

**Screening**

Papers screened on abstract and title.

(n = 2,942)

Papers assessed using global screening.

(n = 74)

**Identification**

Records identified from EMBASE and PubMed:

Databases (n = 2)

Registers (n = 2)

Records removed *before screening*:

Duplicate records removed (n = 491)

Records marked as ineligible by automation tools (n = 0)

Records removed for other reasons (n = 0)

Records excluded

(n = 2,868) and reasons:

- Not related to CVID
- Not related to CVID enteropathy
- No mention of CVID enteropathy histopathology or CVID enteropathy.
- No mention of plasma cells
- Posters
- Primary language was not English or Dutch.
- Review

Excluded papers and reasons(n = 39):

- Paper before 1990
- No qualitative or quantitative description plasma cell content.
- Posters or abstract only.
- Primary language not in English or Dutch.
- No access to full text

Reports assessed for eligibility through full text screening.

(n = 35)

Reports excluded: N = 3:

- Records focused solely on treatment effect.

**Included**

Studies included in review.

(n = 32)
