# Supplementary material for: Paucity of gastrointestinal plasma cells in common variable immunodeficiency
Source: Curr Opin Allergy Clin Immunol. 2024 Oct 7;24(6):464–71. doi: 10.1097/ACI.0000000000001040 (PMC11537466; doi:10.1097/ACI.0000000000001040)
Supplement: Supplementary file 4 [file coaci-24-464-s004.docx]

**Supplementary materials 2:**

We defined our exposure for the ROBINS-E analysis as "absence or paucity of plasma cells", and our outcome to be CVID. Figure 1 shows that all studies correctly account for confounding, as absence or paucity of plasma cells is considered a pathognomonic feature (supplementary figure 1.). Most studies reported their measurement of plasma cell assessment accordingly, with some concerns highlighted in studies that did not specify their choice of immunohistochemistry for plasma cell assessment. In most cases this was a qualitative and not a quantitative methodology. Only one study did not report any methodology of plasma cell assessment.

All studies showed concerns in the selection of participants, as no patients had been selected prior to their diagnosis of CVID or CVID-E. No studies displayed issues due to post-exposure interventions, as no treatment decisions have been made using plasma cell paucity as a metric. Some studies showed severe inconsistencies in their missing data, with mismatch in the number of biopsies assessed per patient being the most common issue. The measurement of the outcome was generally performed as according to the ESID standards; however, some studies did not report the criteria for a CVID. Finally, almost all studies showed some concerns in the selection of reported results, as a pre-determined analysis plan was not available in most cases.
