# Supplementary material for: Paucity of gastrointestinal plasma cells in common variable immunodeficiency
Source: Curr Opin Allergy Clin Immunol. 2024 Oct 7;24(6):464–71. doi: 10.1097/ACI.0000000000001040 (PMC11537466; doi:10.1097/ACI.0000000000001040)
Supplement: Supplementary file 5 [file coaci-24-464-s005.docx]

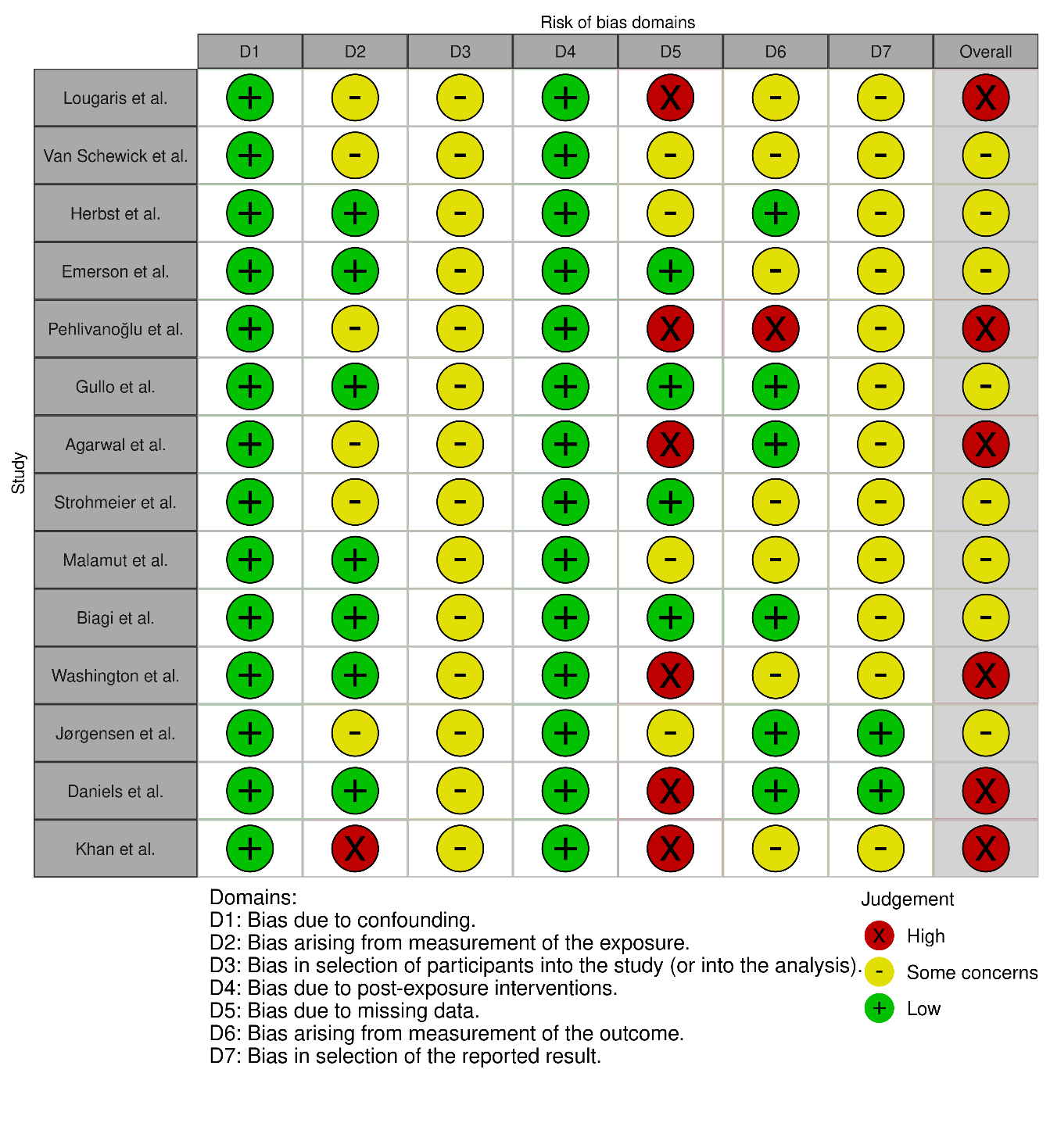


Supplementary figure 1: Stoplight plot highlighting sources of bias stemming from seven domains, ranging from D1-D7. Overall judgement was decided by adhering to the preset guidelines, where the highest risk domain was used to decide overall judgement. Figure generated using the robvis tool provided by the MRC Network of Hubs for Trials Methodology Research.
