# Supplementary material for: Paucity of gastrointestinal plasma cells in common variable immunodeficiency
Source: Curr Opin Allergy Clin Immunol. 2024 Oct 7;24(6):464–71. doi: 10.1097/ACI.0000000000001040 (PMC11537466; doi:10.1097/ACI.0000000000001040)
Supplement: Supplementary file 6 [file coaci-24-464-s006.docx]

Supplementary Table 2: Characteristics of cohorts and locations of biopsies in analysed studies. The type of study performed, the number of biopsies, and locations are all included. Note that not all biopsies and patients were used to assess plasma cell content, therefore, the number of patients and biopsies are inconsistent with table 3 and 4. Studies have multiple and/or metachronous biopsies taken and thus have variable amounts of biopsies per patients are marked with *^±^. ^Δ^* Studies did not differentiate between duodenum, jejunum, and ileum, and so results have been put under duodenum. *N= total biopsies taken or total patients*

| Article: | Study type: | Patients N= 512 | Location biopsies: | | | | | Total biopsies: N= 2239 |
| --- | --- | --- | --- | --- | --- | --- | --- | --- |
|  |  |  | Oesophagus N= 109 | Stomach N= 772 | Duodenum N= 850 | Jejunum + Ileum N= 77 | Colon N= 422 |  |
| Lougaris et al*^±^*^16^ | Retrospective | 22 | 84 | 105 | 84 | - | 30 | 303 |
| Van Schewick et al^± 17^ | Retrospective | 44 | - | - | 46 | 14 | 35 | 95 |
| Herbst et al^22^ | Retrospective | 17 | - | - | 17 | - | - | 17 |
| Emerson et al^23^ | Prospective | 35 | - | 280 | 210 | - | - | 490 |
| Pehlivanoğlu et al^± 26^ | Retrospective | 26 | 5 | 83 | 65 | - | 13 | 172 |
| Gullo et al ^24^ | Retrospective | 9 | - | 9 | - | - | - | 9 |
| Agarwal et al ^29^ | Retrospective | 16 | - | - | 13 | - | 14 | 27 |
| Strohmeier et al ^19^ | Prospective | 65 | - | - | 72 | - | - | 72 |
| Malamut et al^25^ | Retrospective | 50 | - | 200 | 200 | - | 200 | 600 |
| Biagi et al^30^ | Prospective | 17 | - | - | 17 | - | - | 17 |
| Washington et al^±^  ^28^ | Retrospective | 43 | 1 | 9 | 6 | 11 | 8 | 35 |
| Jørgensen et al^± 18^ | Cross-sectional | 53 | - | 50 | 50 | 52 | 52 | 204 |
| Daniels et al*^± Δ 13^* | Retrospective | 20 | 19 | 36 | 39 | - | 35 | 132 |
| Khan et al *^Δ^* ^27^ | Retrospective | 95 | - | - | 31 | - | 35 | 66 |
