# Supplementary material for: Paucity of gastrointestinal plasma cells in common variable immunodeficiency
Source: Curr Opin Allergy Clin Immunol. 2024 Oct 7;24(6):464–71. doi: 10.1097/ACI.0000000000001040 (PMC11537466; doi:10.1097/ACI.0000000000001040)
Supplement: Supplementary file 7 [file coaci-24-464-s007.docx]

*Supplementary Table 3: Study characteristics on Plasma cell content in CVID cohorts. Reporting of the methodology used for the assessment, number of patients and total biopsies, percentages for absence, paucity, and normal are based on total biopsies. – indicates that the study did not use either paucity/reduction or absence as a metric to define plasma cell amounts. ^±^ Noted had metachronous biopsies taken and thus there are variable amounts of biopsies per patient.* ^Ω^ Indicates that study did not include plasma cell analysis in all biopsies, resulting in inconsistencies with table 2. *N= number of total biopsies with absence, paucity/reduction, or normal plasma cells or total patients.*

| Study: | Methodology of plasma cell assessment: | Plasma cell contents in studies as defined through absence, paucity/reduction, and normal: | | | | |
| --- | --- | --- | --- | --- | --- | --- |
|  |  | Total patients N=496 | Total reported biopsies N=671 | Absent  N= 287(43%) | Paucity/reduced N= 151 (23%) | Normal N= 233 (34%) |
| Herbst et al^22^ | In proportion to other mononuclear cells, in percentual difference. Paucity was defined as between >0% - 5%. | 17 | 17 | 4 | 9 | 4 |
| Malamut et al^Ω 25^ | In 10 HPF at 40x magnification and immunohistochemistry. Measured per class. | 50 | 11 ^Ω^ | 7 | - | 4 |
| Emerson et al^23^ | In 10 HPF at 40x where an average of 3 slides were taken. | 35 | 35 | 8 | 13 | 14 |
| Pehlivanoğlu et al^± Ω 26^ | Initial biopsy results, defined as absent or present. | 26^±^ | 165^±Ω^ | 123 | - | 42 |
| Gullo et al ^24^ | Counts per 20x power field, paucity defined as ≤40. | 9 | 9 | 7 | 2 | - |
| Lougaris et al*^±^*^16^ | Present or absent. | 22*^±^* | 21*^±^* | 15 | - | 6 |
| Van Schewick et al^± Ω 17^ | Normal plasma cells, few plasma cells, no plasma cells. | 44^±^ | 83^± Ω^ | 58 | 12 | 13 |
| Jørgensen et al^± Ω 18^ | Paucity or normal. | 53^±^ | 53^± Ω^ | - | 33 | 20 |
| Khan et al ^27^ | Reduced, normal. | 95 | 66 | - | 14 | 52 |
| Strohmeier et al^19^ | Present, absent. | 65 | 58 | 25 | - | 33 |
| Biagi et al^30^ | Present, absent. | 17 | 17 | 15 | - | 2 |
| Washington et al^±^ ^Ω 28^ | Present, absent. | 43^±^ | 26^±^ ^Ω^ | 25 | - | 1 |
| Daniels et al *^±^* ^Ω 13^ | Paucity, normal. | 20^±^ | 110^± Ω^ | - | 68 | 42 |
